# Recent advances in fluorescence anisotropy/polarization signal amplification

**DOI:** 10.1039/d2ra00058j

**Published:** 2022-02-23

**Authors:** Xue Xiao, Shujun Zhen

**Affiliations:** Key Laboratory of Basic Chemistry of the State Ethnic Commission, College of Chemistry and Environment, Southwest Minzu University 610041 Chengdu PR China xiaoxue@swun.edu.cn; Key Laboratory of Luminescence Analysis and Molecular Sensing (Southwest University), Ministry of Education, College of Chemistry and Chemical Engineering, Southwest University 400715 Chongqing PR China

## Abstract

Fluorescence anisotropy/polarization is an attractive and versatile technique based on molecular rotation in biochemical/biophysical systems. Traditional fluorescence anisotropy/polarization assays showed relatively low sensitivity for molecule detection, because widespread molecular masses are too small to produce detectable changes in fluorescence anisotropy/polarization value. In this review, we discuss in detail how the potential of fluorescence anisotropy/polarization signal approach considerably expanded through the implementation of mass amplification, recycle the target amplification, fluorescence probes structure-switching amplification, resonance energy transfer amplification, and provide perspectives at future directions and applications.

## Abstract 2

We discuss how the potential of fluorescence anisotropy/polarization signal approach expanded through mass amplification, fluorescence lifetime amplification, segmental motion amplification, and provide perspectives at future applications.

## Graphical abstract

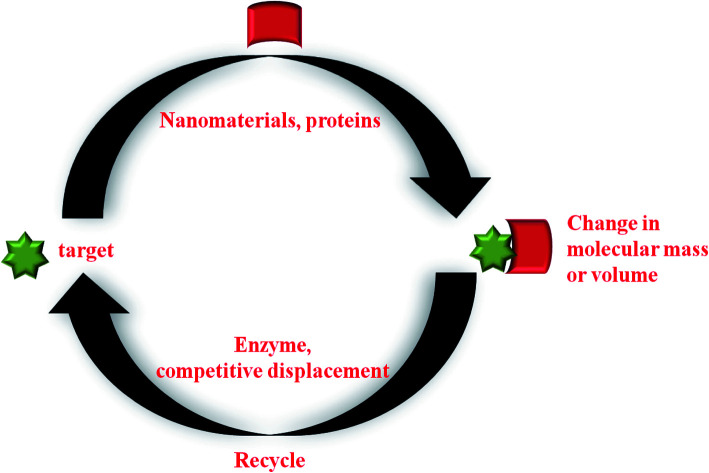

## 1. Introduction

Fluorescence anisotropy/polarization (FA/FP) was first applied in biochemistry almost 7 decades ago when Gregorio Weber described his studies on bovine serum ovalbumin and albumin conjugated with 1-dimethylaminonaphthalene-5-sulfonyl chloride (dansyl chloride).^1–3^ The increase in the number and diversity of FA/FP studies are astonishing and the method is extremely widespread in the clinical and biomedical fields following Weber's work.^3,4^ The utility of FA/FP in clinical and biomedical sciences ultimately rests on the observed polarization on the rotational diffusion rate of molecules.^3^ The reasons for the popularity of FA/FP assays are manifold. First, FA and FP values are intrinsic parameters, and the ratiometric FA and FP techniques are tolerant to photobleaching of the fluorophore and instrumental parameters.^5–8^ Second, FA and FP assays are homogeneous; that is, there is no need for separation of substrates.^3,9^ Third, because of its simplicity and speed, FA/FP assays are especially suitable for an automated high throughput format assay.^10–13^

Earlier FA/FP assays, called fluorescence polarization immunoassays (FPIA), have undoubtedly become predominant methods for the rapid monitoring of small molecules, including abuse of drugs in clinical chemistry and pollutants in environmental and food control.^10,14^ However, the main limitation of FPIA is that the detection of low mass compounds can be fairly restricted.^15–17^ Over the last two decades, the investigation using nucleic acid aptamers as recognition units has broadened the applications of FA/FP assays^18,19^ because of the great stability and high binding affinity of the aptamers and the advantages of aptamers over antibodies in ease of synthesis and labeling.^20^ Different FA/FP assays have been established based on either direct or indirect aptamer recognition system.^20–26^ Likewise, the sensitivities of these strategies are relatively low, because masses of widespread molecules are too small to produce detectable changes in FA/FP value.^27^ Therefore, a general strategy that involves a design for signal amplification will be a highly valued addition to this field. Very recently, the sensitivities of FA/FP assays have been significantly improved by some developed signal amplification approaches such as proteins and nanomaterials mass amplification,^28–33^ enzyme-catalyzed recycling signal amplification,^27,34^ competitive displacement and induced-fit binding amplification.^35,36^

Previous reviews that tackled the FA/FP-based sensing platforms mainly focused on the specific recognition elements and its applicative aspects.^3,4,6,16,17,37^ Qiu *et al.* summarized about nanomaterial enhanced FP/FA technology,^38^ however, there is currently no thorough review dedicated to design for signal amplification about FP/FA. Instead, the present feature review aims at thorough discussing the state of art in signal amplification mechanisms and methods design, with a special emphasis on their analytical performances and implications in terms of scope.

## 2. The principle of FA/FP assay

The basic principle of measurements relies on the absorption of linearly polarized excitation light by a fluorophore in relation to its orientation.^16^ The polarization (*P*) and anisotropy (*r*) terms, both depending on the measured emission intensities parallel and perpendicular to the plane of the vertically polarized excitation light, can interchangeably be used for determining the polarization degree of a fluorescent rotating species.^3,15,39^ At the molecular level, *P*/*r* is related to the rotational correlation time (*θ*) and the fluorescence lifetime (*τ*) of fluorescent molecules, and can be expressed by the modified form of the Perrin formula:1
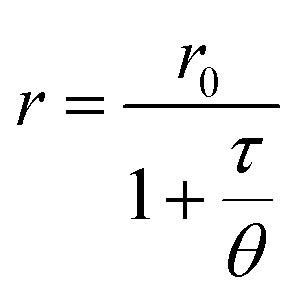
where *r*_0_ is the fundamental FA, that is the anisotropy in the absence of rotation. *θ* is related to the diffusion coefficient, which in turn depends on the solvent viscosity (*η*), solution temperature (*T*) and the volume of the rotating spherical species (*V*). Therefore, the following relation can be obtained:2
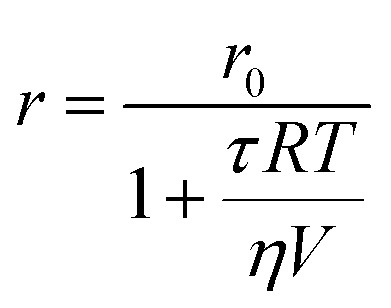
where *R* is the gas constant. According to eqn (2), for a fixed excitation wavelength and a constant *τ*, and in the absence of any additional phenomena, an increase in the apparent molecular volume (or in the molecular mass) of the fluorescent species will produce high polarization.^16^

FA or FP, observed when fluorophores are excited by polarized light, is a commonly measured property of fluorescent molecules.^6^ The FA values (*r*) can be readily obtained by determining the fluorescence intensities from two polarization planes, normally, calculated using the following eqn (3):3
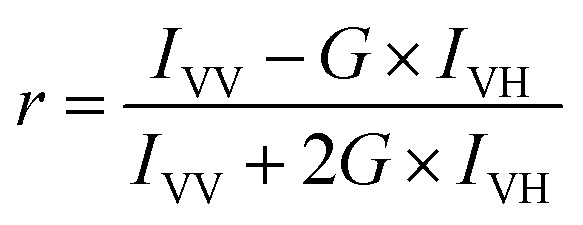
and4
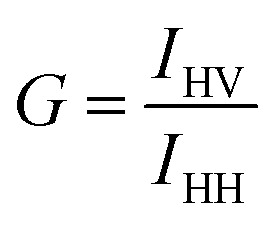
where *I* is the fluorescence intensity, and the subscripts V and H refer to the orientation (vertical and horizontal respectively) of the polarizer. The first subscript indicates the position of the excitation polarizer, while the second indicates the position of the emission polarizer. *G* is the instrumental correction factor, which corrects for the different detection efficiencies of horizontal and perpendicular emission pathways.

According to eqn (5), the anisotropy (*r*) and polarization (*P*) values are interchangeable. As a measure of the ratio of intensities, the anisotropy or polarization is a dimensionless value. In principle, the anisotropy and polarization are independent of the fluorophore concentration and the total intensity of the sample.^6^5
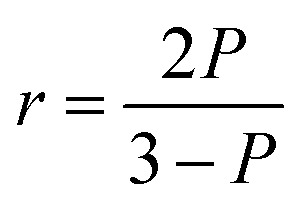


## 3. Strategies without signal amplification

FA/FP assays can be employed for protein detection under a direct format, because of the relatively large mass or volume of protein. In these approaches, fluorescent dye modified aptamer has been used as FA/FP probe and target recognizing unit. When protein analyte is added, an effective increase in the mass of the aptamer probe occurs upon complex formation, resulting in detectable variations in their FA values. However, most of the methods commonly suffer from poor FA responses, ranging from 0.050 to 0.100.^40–43^

FA is not only influenced by the molecular weight of the formed complex, but also in relation to the nature of the tethered fluorophores.^20,44^ For example, the electrostatic interaction between the dye and DNA affects the segmental motion of dye, so the choice of the charge characteristics of the fluorophore is crucially important.^45^ Taking advantage of the electrostatic interaction between fluorescein and lysozyme, Li group developed a simple and sensitive analytical method for lysozyme.^46^ In this contribution, a coupling of negatively charged fluorescein with positively charged lysozyme upon binding between lysozyme and its aptamer occurs. Such coupling may bring an increased fraction of global rotational movement of the fluorescence species, thus producing a significantly increased FA signal with the maximum changed value of 0.2. However, this method can be only applied to peculiar fluorophore/protein pairs of opposed charge.

**Fig. 1:**
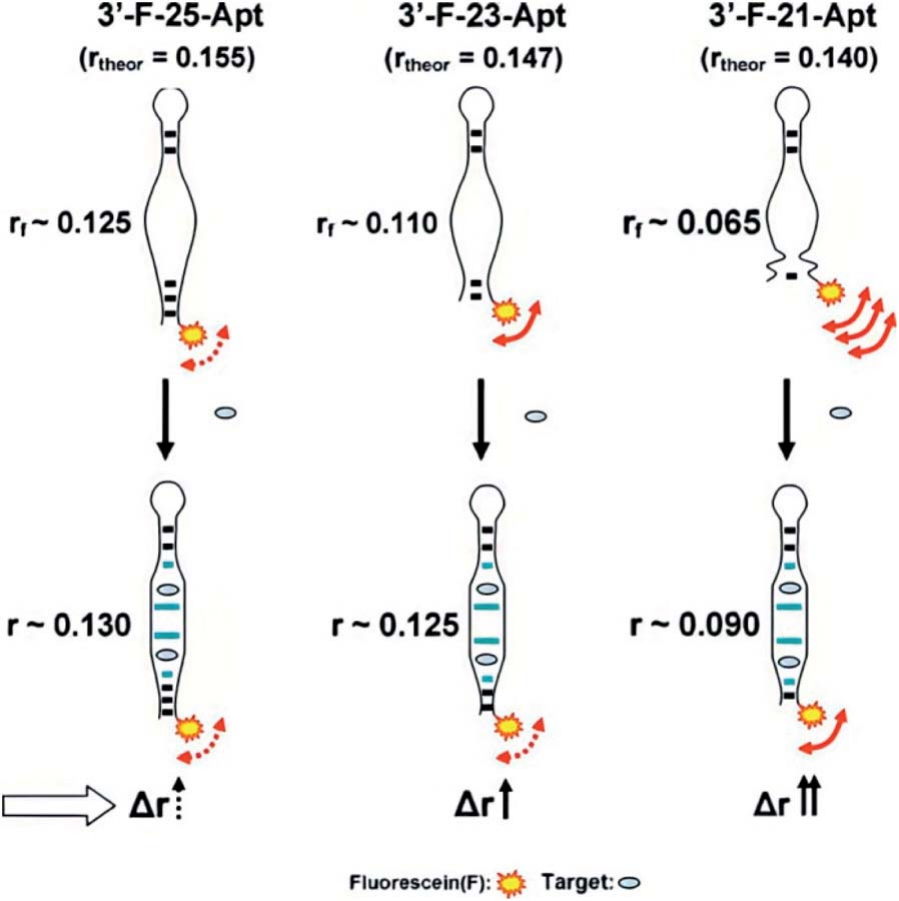
Proposed representation of the local segmental motion of dye contributed (arrow) to the variation of the FA signal in relation to the aptamer probe structure. *r*_f_ is the FA in the absence of analyte. Reprinted with permission from ref. 26. Copyright (2010) Elsevier.

In addition, small molecules and metal ions also have been analyzed by a direct FA/FP format.^22,24,47–49^ Likewise, the FA/FP variation experimentally observed is somewhat negligible. Through changing the structure of aptamer and the coupling mode of aptamer and fluorescent dye, some studies have been developed to overcome this constraint. Peyrin group designed an engineering instability in the secondary structure of an aptameric recognition element that is usually a well-defined stem-loop secondary structure (Fig. 1).^26^ With this method, the segmental mobility of a fluorophore label, attached to one extremity of the aptamer, can be significantly promoted so that a strong depolarization process will occur. Upon target binding, the structural change of the aptamer will favor the formation of a stable stem-loop structure, inducing an increase in the FA signal. However, this strategy is not widely applicable as it requires a sufficiently large aptamer conformational/structural change or the rational engineering of the aptameric element. Furthermore, taking advantage of competitive reaction between complementary sequences of aptamer and small molecules, their group exploited the small molecules analytical methods with broader applicability.^23,50^ Zhao *et al.* utilized intramolecular tetramethylrhodamine (TMR) labeling of ssDNA to achieve a noncompetitive FA assay for the detection of small molecules.^20^ The intramolecular interaction between labeled TMR and DNA aptamer bases dramatically affects the local rotation and FA of TMR and can be altered by aptamer conformation change upon target binding, leading to a significant change of FA of TMR.

Rational design of the linker between fluorophores and target identification unit, appropriate choice of fluorophores, contribute to increase sensitive FA/FP responses. Even so, all these direct size-based assays exhibit modest FA/FP change and sensitivity. Direct assays have the advantage of simple reaction, but its application scope subject to certain limitations, especially for the detection of small molecules and metal ions as they are too small to induce the obvious FA changes.

## 4. Strategies with signal amplification

To enhance FA/FP response, substantial efforts were devoted to the development of signal amplification methodologies. There are mainly creations in two aspects: one is to label the fluorophore with large molecules or nanomaterials, such as proteins, gold nanoparticles (AuNPs), SiO_2_ nanoparticles (SiO_2_NPs), or graphene oxide (GO); the other one is to design a specific system to recycle the target molecules, such as enzyme-catalyzed target recycling, target-catalyzed DNA cyclic assembly. Next, we discuss these methods.

### 4.1 Protein amplification strategies

**Fig. 2:**
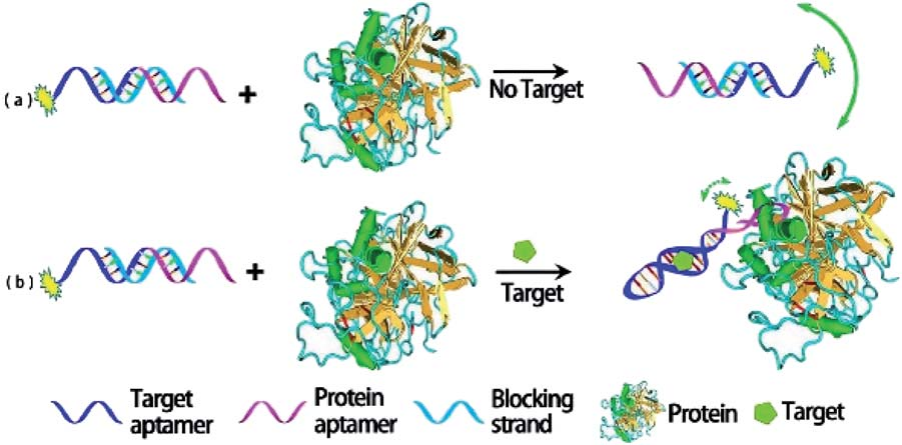
Working principle of protein amplifying FA for sensitive detection of small molecules. Reprinted with permission from ref. 28. Copyright (2012) American Chemical Society.

Proteins, which are larger molecular mass or volume than aptamers, can be used to enhance FA/FP signal.^51^ Famulok *et al.* used protein-binding aptamers for screening small molecule inhibitors of proteins in an FA/FP approach.^52^ In addition, Yang group developed a protein mass amplifying strategy to construct FA aptamer probes for small molecule analysis in complex biological samples.^28^ In this approach (Fig. 2), a mass amplifying probe consists of a targeting aptamer domain against a target molecule and a molecular mass amplifying aptamer domain for the amplifier protein. The probe is initially rendered inactive by a small blocking strand, and will be activated to be bound to the protein only when a target molecule is bound to the probe. Enlarging the molecular mass of the probe/target complex can obtain an obviously increased FA value. In this effect, two probes that constitute a target (ATP and cocaine respectively) aptamer were prepared, and the detection limits for ATP and cocaine were 0.5 μM and 0.8 μM, respectively.

Significantly, streptavidin as a large-sized protein has been employed as an effective signal amplifier to detect small molecules^49,53^ and analyze the DNA–protein interactions.^54^ Unlike other FA/FP signal amplification strategies that use dual binding DNA probes, these methods implement signal amplification through the binding of streptavidin with biotinylated DNA.^54^ Moreover, to further improve the sensitivity of detection, various signal amplification strategies combining with streptavidin mass amplification have been developed. First, hybridization chain reaction (HCR) and polymerase chain reaction (PCR) was used to construct a target-triggered assembly of DNA–protein hybrid nanowires for detection of small molecules and chloramphenicol respectively.^12,55^ Second, combining target-triggered enzymatic cleavage protection and the extraordinarily strong interaction between biotin and streptavidin, a novel mass amplification strategy for the sensitive detection of adenosine^56^ and Pd^2+^ (ref. 57) in homogeneous solution was reported. Third, Dzantiev *et al.* developed an FP-based aptamer assay with the use of anchor protein modules, based on the streptavidin and IgG as FP enhancers.^51^ The change of FP which was dually enhanced by streptavidin and IgG respectively could be used to detect ochratoxin A at the nM level in wine within 15 minutes.

To sum up, FA/FP assay response and sensitivity are improved by protein amplification. However, the limitation for use of protein amplifiers in FA/FP analysis is constituted by their variabilities and the need to operate under precisely defined experimental conditions compatible with the properties and stability of biomolecules.^16^

### 4.2 Nanomaterials amplification strategies

Compared with proteins, nanomaterials amplification have high thermal stabilities, easy synthesis and modification, and the FA/FP signal change is more significantly improved.^58^ Recently, to improve the sensing sensitivity, a number of nanomaterials such as AuNPs, CNTs, GO, metal–organic frameworks (MOFs), SiO_2_NPs, quantum dots (QDs), 2D transition metal dichalcogenide nanosheets (TMD NSs), were introduced into FA/FP sensor system. Nanomaterials as FA/FP enhancer has brought revolutionary effect on greatly improving detection sensitivity and application range of FA/FP because of its unique structure and extraordinary properties such as simple/controllable synthesis and modification, different sizes, large volumes and masses, excellent optical and chemical properties.^38^ Furthermore, several nanostructures were specifically bound to ssDNA, such as GO, MOFs and MoS_2_, giving discriminating effects to the free, unstructured and bound, structured states of a functional nucleic acid. This unique nucleic acid interaction forms the basis for a versatile mass amplifying strategy for the FA/FP technique.^31^ However, the amplification strategies that utilize nanomaterials are usually compromised by fluorescence quenching and scattering, potentially providing a less accurate measurement.^16,54^ Moreover, the difficulty to apply to living cells or organisms because of their widespread biotoxicity constitutes another limiting factor.

#### 4.2.1 Gold nanomaterials amplification strategies

**Fig. 3:**
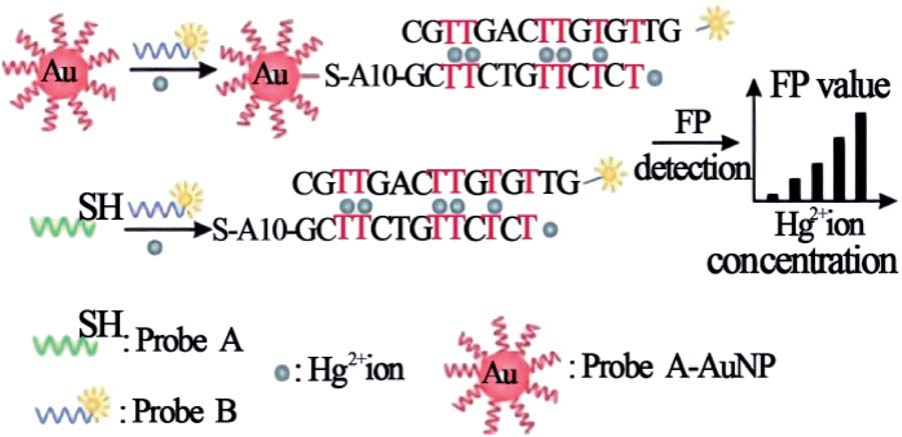
Schematic illustration of the strategy of Hg^2+^ ion detection using FP enhancement by AuNPs. Reprinted with permission from ref. 65. Copyright (2008) Wiley.

Gold nanomaterials have widespread applications in biological studies due to their huge specific surface area, unique optoelectronic properties, ease of synthesis, excellent stability and low cytotoxicity.^59,60^ There are a large number of studies that have demonstrated well that gold nanomaterials can substantially improve the performance and sensitivity of different biosensing systems.^61–64^ The first application of gold nanomaterials amplification was described for the Hg^2+^ detection.^65^ In this strategy, Ye *et al.* used AuNPs to increase the volume or mass of the fluorescence entities in fluorescence anisotropic determination of mercury ion by the formation of T–Hg^2+^–T mediated structure. Two DNA probes that contain six strategically placed thymine–thymine mismatches complexed with Hg^2+^ ions were used, one labeled with a fluorophore, and the other conjugated onto the surface of AuNPs. As shown in Fig. 3, the stable hybridization between probe A and probe B occurs only when Hg^2+^ ions are present, releasing probe A from AuNPs, causing a large change in molecular size and anisotropy. This method achieved a detection limit of 1 nM for Hg^2+^.

Furthermore, Ye group also employed a novel and practical method relying on metal ion-dependent DNAzyme-based FA *via* AuNPs enhancement, which was presented for detecting Cu^2+^ and Pb^2+^ ions in an aqueous medium at room temperature.^61^ Ren group used AuNPs as a dual-enhancer of FP for nucleic acid detection.^66^ In this report, AuNPs increased the volume or mass of the fluorescent dye Alexa fluor 488 dyes (Alexa488) and the AuNPs-mediated nanometal surface energy transfer (NSET) quenching effect decreased the effective concentration of the Alexa488, which caused remarkable dual enhancement of the Alexa488's FP.

#### 4.2.2 Silver nanoparticles amplification strategies

**Fig. 4:**
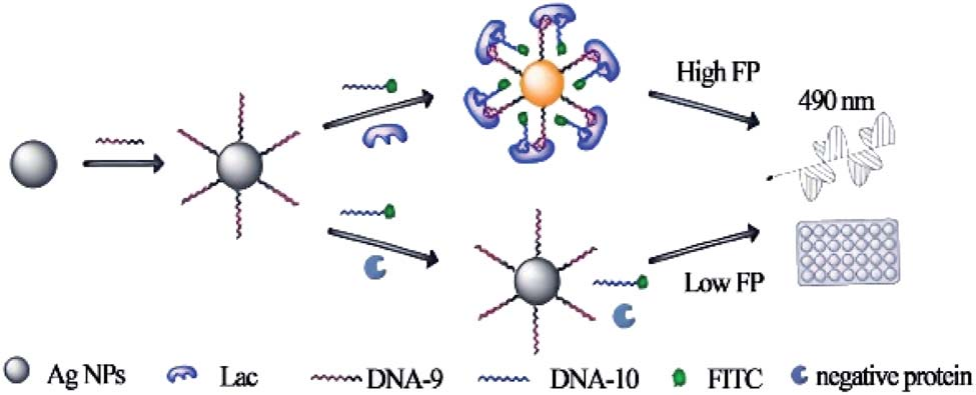
Principle of the dual amplified aptasensor based on bivalent aptamers and Ag_10_NPs enhancement. Reprinted with permission from ref. 68. Copyright (2017) American Chemical Society.

Silver nanoparticles (AgNPs) are of particular interest in biotechnology owing to their intense optical properties based on the surface plasmon resonance, electrochemical and catalytic activity, and strong anti-microbial activities. Few have reported the use of AgNPs in FA/FP comparing with AuNPs. Zhao *et al.* first introduced AgNPs to the FP system, inventing a mass-augmented FP method for both Hg^2+^ and cysteine detection.^67^ In addition, Xu group reported a bivalent aptasensor based on silver enhanced FP for rapid detection of lactoferrin (Lac) in milk.^68^ This strategy employed a dual recognition of the aptamers that have been split into two parts and the metal-enhanced fluorescence (MEF) effect of Ag_10_NPs on signal molecule fluorescein isothiocyanate (FITC). As shown in Fig. 4, the sequence of bivalent aptamer was innovatively split into two fragments. One was assembled on the surface of Ag_10_NPs and the other one was labeled with FITC dye. In the absence of target protein, the split aptamers labeled with FITC dye exhibited a relatively low FP value due to their smaller size. However, upon recognizing and binding with protein, FP values increased greatly as the structure of split aptamer favored the formation of a stable split aptamers-target−Ag_10_NPs complex, inducing the reduction of FITC's mobility and MEF effect, thus causing the increase of FP signal. This design achieved the ternary signal amplification, including bivalent aptamers amplification, MEF effect amplification and Ag_10_NPs amplification, thus showed a highly sensitive response to Lac with the detection limit of 1.25 pM.

#### 4.2.3 Carbon nanomaterials amplification strategies

Carbon nanomaterials amplifying FA detection strategy is mainly based on the difference of binding affinity of carbon nanomaterials with ssDNA and the DNA-target complex. Carbon nanomaterials have strong binding ability with ssDNA through π–π stacking interactions, but the affinity of the DNA-target complex is significantly weaker than that of ssDNA.^31,69–73^ Furthermore, compared with AuNPs and AgNPs, they require neither modifying the surfaces of nanoparticles nor sophisticated probe design. Carbon nanomaterials' large volume or mass, in combination with the unique DNA interactions, form the basis for a convenient and versatile mass amplifying strategy for the FA technique.^31,58^ Nonetheless, one drawback for the use of carbon nanomaterials is the competitively releasing the DNA tracer from the carbon nanomaterials surface because of the intense π–π stacking and electrostatic interactions.

A multiwalled carbon nanotube as a signal amplifier for FP assay of DNA methyltransferase (MTase) activity has been developed.^74^ In this work, the introduction of a multiwalled carbon nanotube causes a significant amplification of the detection signal, which substantially improves the detection sensitivity by two orders of magnitude over the reported methods. In addition, by using carbon nanoparticles to enhance FA, an aptamer-based sensor enabling signal-amplification and real-time detection of apyrase is reported.^75^

Significantly, GO as a two-dimensional (2-D) nanosheet with facile synthesis and high water dispersity, contributes to a slower rotation than that of spherical nanoparticles with the same surface area.^76,77^ Thus, GO is able to perform as an excellent FA amplifier. Yang *et al.* reported a FA signal amplification strategy by employing GO as the signal amplifier to detect adenosine triphosphate (ATP).^31^ Huang and coworkers developed a GO amplifying strategy to construct a DNAzyme-based FA system for Cu^2+^ analysis.^58^ Huang group also developed a label-free FA method by using GO as FA amplifier, G-rich single ssDNA as recognition probe, acidine orange as FA reporting fluorophore and potassium ion (K^+^) as a proof-of-concept target.^33^ This contribution does not require covalent modification of the recognition probe, thus it is simple and cost-effective. GO-amplified FP assays also were used for the identification of antagonists^78^ and toxins.^79,80^ In addition, to further improve the sensitivity of the GO-enhancer FA/FP method, exonuclease and nicking enzymes have been employed to achieve target recycling for signal amplification detection.^81,82^

**Fig. 5:**
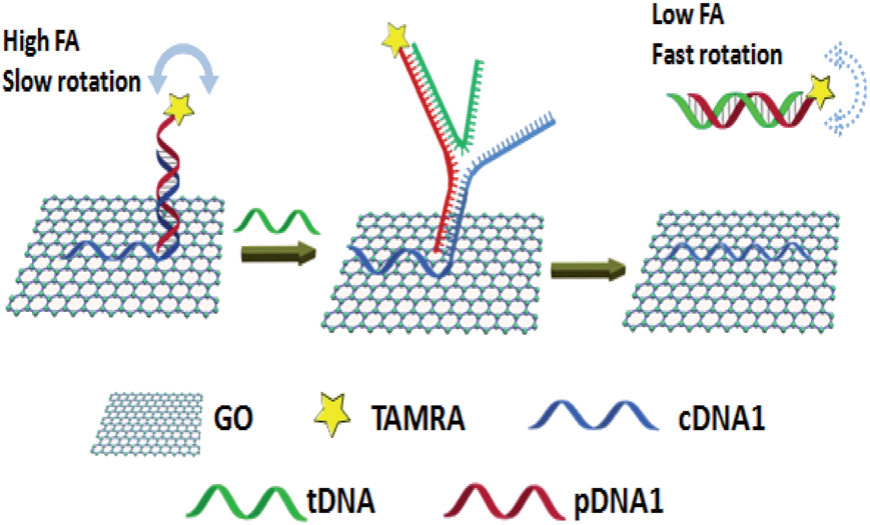
A schematic representation of ssDNA detection using a novel GO amplified FA assay. Reprinted with permission from ref. 9. Copyright (2015) Royal Society of Chemistry.

Especially, for decreasing the quenching efficiency of GO to the fluorescence of dye-labeled probe DNA to ensure FA detection accuracy, Zhen group reported a novel GO amplified FA assay with improved accuracy and sensitivity.^9^ In this approach (Fig. 5), a toehold-mediated strand exchange reaction was introduced to detach probe DNA from the GO surface. The probe DNA was indirectly immobilized on GO through the double-stranded region of its hybridization with a capture DNA fragment that contained an A_20_ tail. Through this design, not only the rotation of fluorescent dye was restricted, but also the quenching efficiency of GO to fluorescent dye was decreased. And in the presence of the target, probe DNA was released from the GO surface more easily through toehold-mediated strand exchange reaction comparing with the method in which probe DNA is directly immobilized on GO, resulting in obviously decreased FA. This strategy has been used for the selective detection of ssDNA, adenosine and thrombin. Besides, based on the above mechanism, to enhance the detection sensitivity, Zhen and coworkers also employed the exonuclease III (Exo III) and target-catalyzed hairpin assembly (CHA) to achieve recycling signal amplification.^27,83^

#### 4.2.4 SiO_2_NPs amplification strategies

**Fig. 6:**
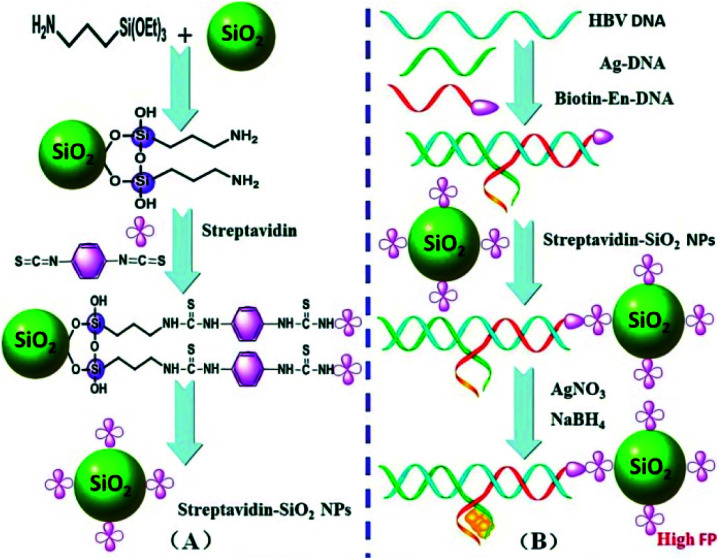
(A) Synthesis procedure for the functionalized streptavidin–SiO_2_NPs. (B) A working principle of the proposed FP biosensor for HBV DNA detection. Reprinted with permission from ref. 85. Copyright (2015) Royal Society of Chemistry.

SiO_2_NPs as a biocompatible and versatile substrate for probe immobilization has been exploited to enhance FP signal. Nonetheless, they usually require functionalizing the surfaces similar to AuNPs. Liang *et al.* reported an FP aptasensor that relied on aptamer structure-switching-triggered SiO_2_NPs enhancement for ATP detection.^84^ Qiu group developed a novel versatile FP platform based on the SiO_2_NP-DNA/Ag nanocluster (NC) sandwich structure as a signal enhancer for optical detection of HBV DNA in biological media.^85^ As depicted in Fig. 6, functionalized streptavidin–SiO_2_NPs linked the En-DNA probe which was tagged at the 5′-terminus with a biotin molecule through the noncovalent biological interaction of streptavidin at the surface. In the presence of target HBV DNA, the sandwich structure will be formed at the surface of SiO_2_NPs resulting in a substantial increase of the FP value due to the enlargement of the molecular volume of the formed SiO_2_NP-functionalized DNA/Ag NC sandwich structure. Thus, the detection of target HBV DNA can be easily realized by monitoring the increased FP values. This method does not require covalent labeling fluorescent dye of the recognition probe, so it is very simple and cost-effective. In addition, a double amplified FP assay established based on HCR and SiO_2_NPs combination *via* biotin–streptavidin interactions has been reported, which makes a large molecular weight enhancement and enables simple, sensitive and selective detection of nucleic acid.^86^ Moreover, combining with the click chemistry, Zhao *et al.* developed a SiO_2_NPs-assisted FP enhancement method for the sensitive detection of Cu^2+^.^87^ In this work, Cu^+^-catalyzed azide–alkyne cycloaddition (CuAAC) reaction which is a typical example of “Click Chemistry” was as chemically ligase DNA chains, the high specificity of that improved the selectivity of detection. This approach achieved a detection limit of 0.0178 μM for Cu^2+^.

#### 4.2.5 Metal–organic nanomaterials amplification strategies

**Fig. 7:**
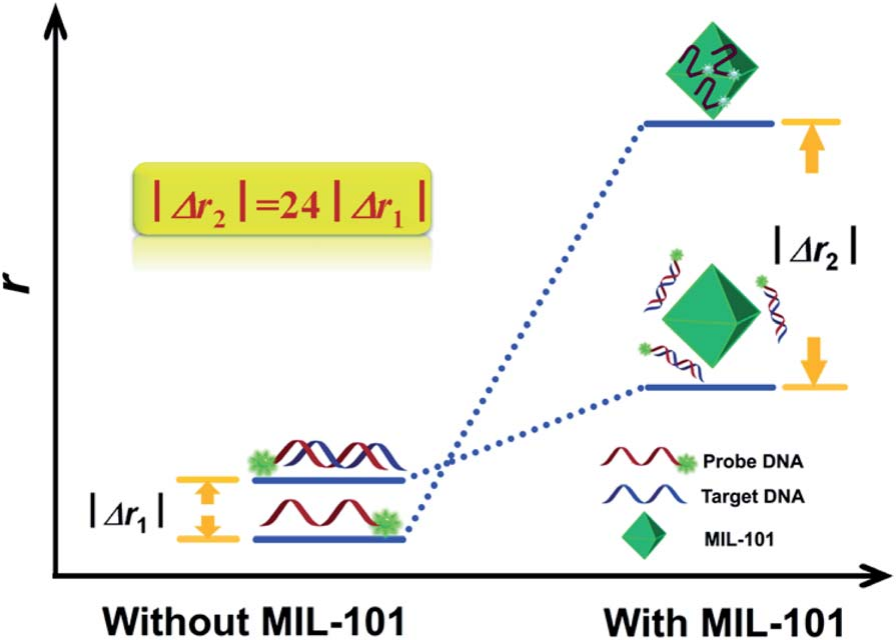
The concept and the principle of MIL-101 amplified fluorescence anisotropy strategy for HIV-DNA detection. Reprinted with permission from ref. 32. Copyright (2014) Royal Society of Chemistry.

Metal–organic frameworks (MOFs) that combine metal ions with rigid organic ligands, usually have large molecular masses and volumes, supplying a potential ability of higher enhancement efficiency of FA/FP. Li group explored a series of analytical applications of MOFs amplification FA signal. Chromium-benzenedicarboxylates (MIL-101), one of the cationic MOFs, have strong affinity to negatively charged DNA through π–π stacking and electrostatic interaction. Its mechanism is similar to the interaction between GO and biomolecules. First, MIL-101 was introduced as a FA amplifier for the detection of ssDNA.^32^ In this contribution, as shown in Fig. 7, probe DNA was absorbed and twined onto the surface of MIL-101 before adding a target DNA, exhibiting a large FA value. Upon addition of target DNA, the formed dsDNA of probe DNA/target DNA kept away from MIL-101, resulting in a significant change in FA value. Second, a logic gate assay based on MIL-101 as an FA enhancer for the detection of mercury and iodide ions can be constructed.^88^ This is the first time that an approach introduces FA as the output signal of logic gate, expanding the application of FA and MOFs. Third, a dual amplifying FA assay for label-free detection of respiratory syncytial virus DNA fragments with size-control synthesized MIL-101 has been reported.^89^ Moreover, metal–organic gel (MOG), composed by the self-assembly of metal ions and suitable ligands through noncovalent interactions with the same feature as MOFs that are able to adsorb ssDNA, was also exploited as a novel amplification platform for FA assay by Li's group.^90^ This strategy achieved a sensitive detection of a common cancer biomarker, prostate specific antigen (PSA).

#### 4.2.6 Transition metal nanomaterials amplification strategies

**Fig. 8:**
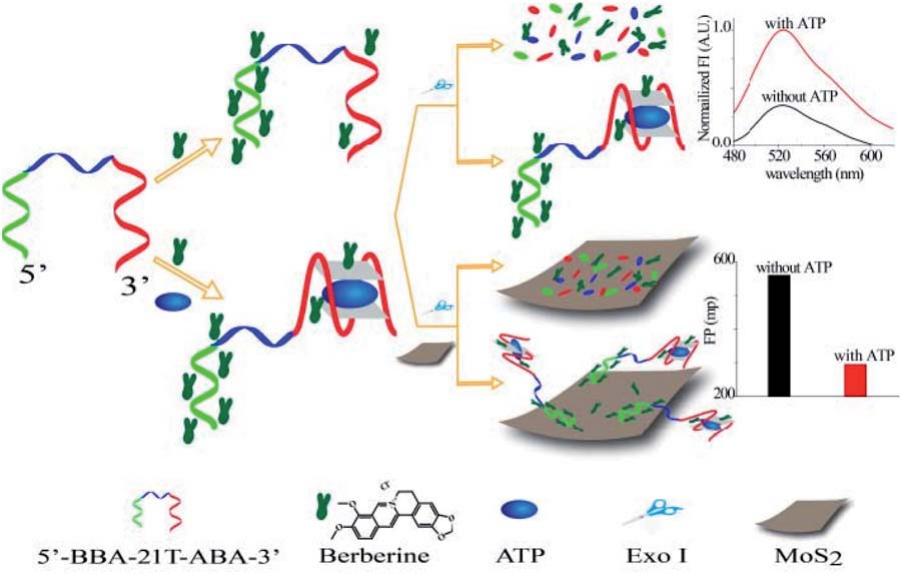
Illustration of a chimeric aptamers-based and MoS_2_ nanosheet-enhanced label-free FP strategy for ATP detection. Reprinted with permission from ref. 94. Copyright (2018) American Chemical Society.

2D transition metal nanosheets with single- and few-layers, such as WS_2_, MnO_2_, TiS_2_ and MoS_2_, have attracted great attention due to their unique electronic, optical and electrochemical properties, exhibiting many promising applications in the fields of electronics, sensors, catalysis, and energy storage. In recent years, transition metal nanomaterials have been exploited as FA/FP amplifier. First, WS_2_ nanosheet as an amplifier and a scaffold to develop amplified FP bioassays is reported.^91^ Second, Zhang's group designed a MnO_2_ nanosheet-assisted ligand–DNA interaction-based fluorescence polarization method for sensitive detection of Ag^+^.^92^ This study utilized proflavine as the indicator does not suffer from limitations owing to unnatural folding and the formation of biomacromolecular structures. Third, Fan' group developed a TiS_2_ nanosheet enhanced FP biosensor for the detection of folate receptor and thrombin.^93^ Moreover, Zhang developed a MoS_2_ nanosheet-enhanced FP aptasensor for ATP detection.^94^ In this strategy, a bifunctional DNA strand was designed to consist of chimeric aptamers that recognize and capture ATP and berberine. As depicted in Fig. 8, in the absence of ATP, the DNA strand bound to berberine will be hydrolyzed when Exonuclease I (Exo I) is introduced, releasing berberine. And then, released berberine combined with MoS_2_, generating a substantial increase of the FP value. On the contrary, when ATP is present, ATP aptamer folds into a G-quadruplex structure, thus the complex can resist degradation by Exo I to maintain berberine far away from MoS_2_, resulting in a low FP value. This nanosheets-enhanced FP strategy is simple and facile which does not require traditional dye-labeled DNA strand showed a high sensitivity for the quantification of ATP with a detection limit of 34.4 nM.

#### 4.2.7 DNA nanosheet amplification strategies

Various inorganic nanomaterials have been used as FA/FP enhancers, however, most of them are size-uncontrollable and possess an intensive fluorescence quenching ability, which reduced the accuracy and sensitivity of the FA/FP method. Zhen group reported a two-dimensional DNA nanosheet (DNS) without fluorescence quenching effect as a FA amplification platform for the detection of ssDNA, ATP and thrombin.^95^ This method improved the accuracy of FA assay and proved that DNA nanosheet is an excellent FA amplification nanomaterials.

#### 4.2.8 Polystyrene nanomaterials amplification strategies

Polystyrene nanomaterials have also been developed as FA/FP enhancers for biomolecules detection. Liang *et al.* exploited an amplified FP aptasensor based on polystyrene nanoparticle (PS NP) enhancement and allostery-triggered cascade strand-displacement amplification (CSDA) for ultrasensitive detection of proteins.^96^ In this study, PS NP as mass enhancer connected DNA duplex probes *via* the streptavidin–biotin binding. Combining with the CSDA process, numerous massive dyes are assembled onto PS NPs, and achieve a cyclic amplification signal, which results in a substantial FP increase. This strategy has a wide detection range of up to 6 orders of magnitude and a detection limit of nearly 6 orders of magnitude lower than that of traditional homogeneous aptasensors. Furthermore, based on polystyrene nanospheres and T7 exonuclease assisted dual-cycle signal amplification, Zhao *et al.* reported a sensitive fluorescence polarization method for the detection of biomarker microRNA (miRNA)-141.^97^ And this method was also applied to detect and compare the expression level of miRNA-141 in different cells.

### 4.3 Enzyme amplification strategies

**Fig. 9:**
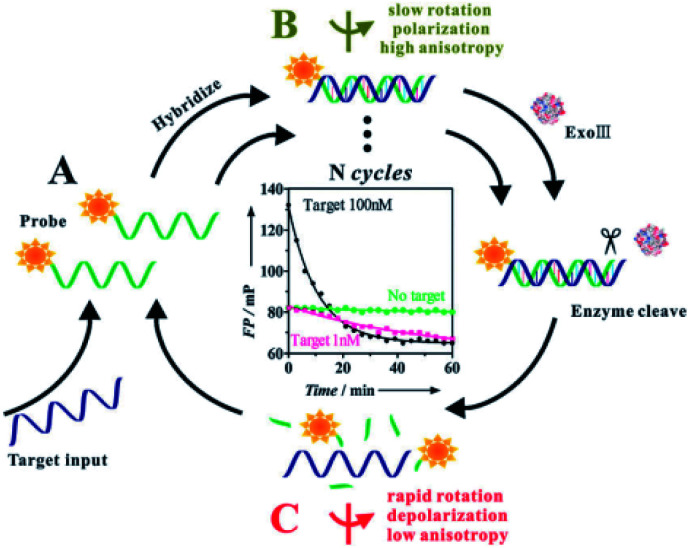
Target assisted Exonuclease III-catalyzed amplification FP for DNA detection. Reprinted with permission from ref. 34. Copyright (2011) Royal Society of Chemistry.

The strategies based on the enzyme-catalyzed target amplification have demonstrated great potential. In FA/FP assay, there are two main types of enzyme amplification signal, cleavage enzyme and polymerase. First, Ye group reported a versatile target assisted Exo III-catalyzed amplification FP methodology for the highly sensitive and selective detection of DNA.^34^ In this contribution, Exo III is employed to recycle the process of target-assisted digestion of probe molecules, thus resulting in significantly improved sensitivity. In the presence of the target DNA, the hybridization of the target and probe strands form a double-stranded structure containing a blunt 3′ terminus and an Exo-III-resistant 3′ protruding terminus (Fig. 9, B). Exo III can catalyze the stepwise removal of mononucleotides from 3′-hydroxyl termini of duplex DNAs with blunt or recessed 3′ termini. The target–probe hybridization triggers the selective enzymatic digestion of the dye-labeled probe, liberating the fluorophore with several nucleotides (low fluorescence polarization value) before ultimately releasing the target (Fig. 9, C). The released intact DNA target then hybridizes with another dye-labeled probe to initiate the cycle digestion of the probe, resulting in the digestion of many probes, generating a substantial decrease of the FP value. In addition, Lee group developed an FP approach for nucleic acid tests that used DNA polymerase to accomplish reverse transcription (RT-) PCR and recycle for the extension reaction on the reporter probes.^98^ As we mentioned above, different enzymes combined with nanomaterials amplification techniques have been proven to be increasingly valuable for quantitative analysis of various target molecules, such as nicking enzyme assisted GO-based dual signal amplification,^82^ T7 exonuclease assisted polystyrene nanospheres dual-cycle signal amplification.^97^ Moreover, enzymes combined with protein amplification FA/FP assays have been exploited to the sense of small molecules.^56,99^

### 4.4 Target-catalyzed DNA cyclic assembly amplification strategies

**Fig. 10:**
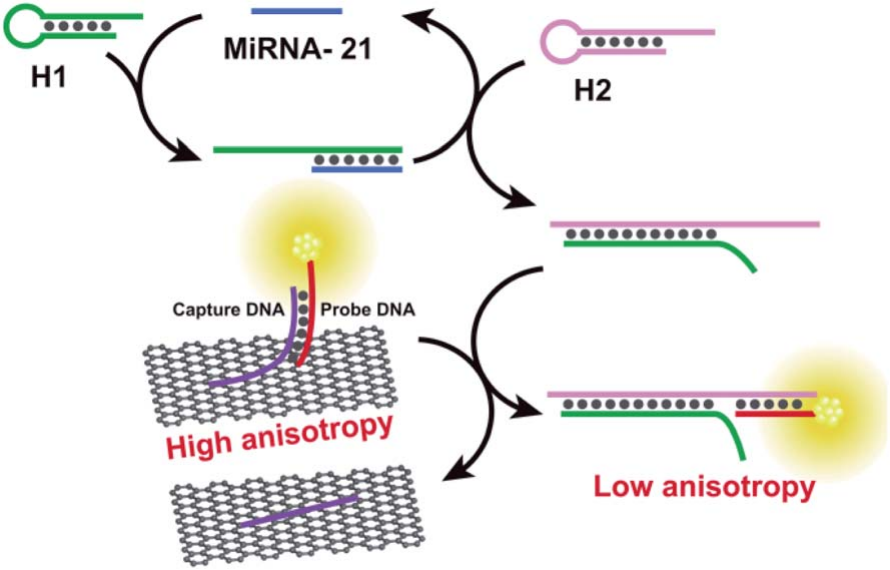
Schematic illustration of enzyme-free FA assay for miRNA-21 detection. Reprinted with permission from ref. 83. Copyright (2017) American Chemical Society.

The protein enzymes usually can achieve cyclic amplification FA/FP signal. However, as they can be denatured and digested by proteases easily, it is hard to operate them in complex biological samples.^100,101^ Enzyme-free signal amplification strategies based on toehold-mediated DNA strand displacement have been developed to amplify FA/FP signal, which works under mild conditions without specific demand for either ionic strength or temperature comparing with enzyme amplification. Tan group reported a target-triggered assembly approach based on the formation of DNA–protein hybrid nanowires *via* HCR, using ATP as a small molecule model.^12^ This novel dual-amplified, aptamer-based FA assay affords high sensitivity with a detection limit of 100 nM for ATP. Zhen *et al.* developed the new target-catalyzed hairpin assembly (CHA), enzyme-free DNA circuit, and assisted GO amplified FA strategy for miRNA detection.^83,102^Fig. 10 depicts the principles of CHA assay. The fluorescent dye, carboxytetramethylrhodamine (TAMRA), modified probe DNA was first hybridized with capture DNA containing an A_20_ tail. This design can not only improve the sensitivity of FA detection but also can decrease the fluorescence quenching effect of GO, thus ensuring the accuracy of FA detection.^9,27^ In the presence of miRNA-21, the CHA was initiated and plenty of H1–H2 duplexes were produced continuously. The obtained H1–H2 duplex could trigger the toehold-mediated strand exchange reaction to form an H1–H2-probe DNA complex that detached the dye-modified probe DNA from the GO surface, leading to a decreased FA of the system. The detection limit of this method was 47 pM, which was 279 times lower than that of the method without CHA. And the selectivity of this method has also been enhanced greatly compared with the method without CHA.

### 4.5 Oligonucleotides amplification strategies

**Fig. 11:**
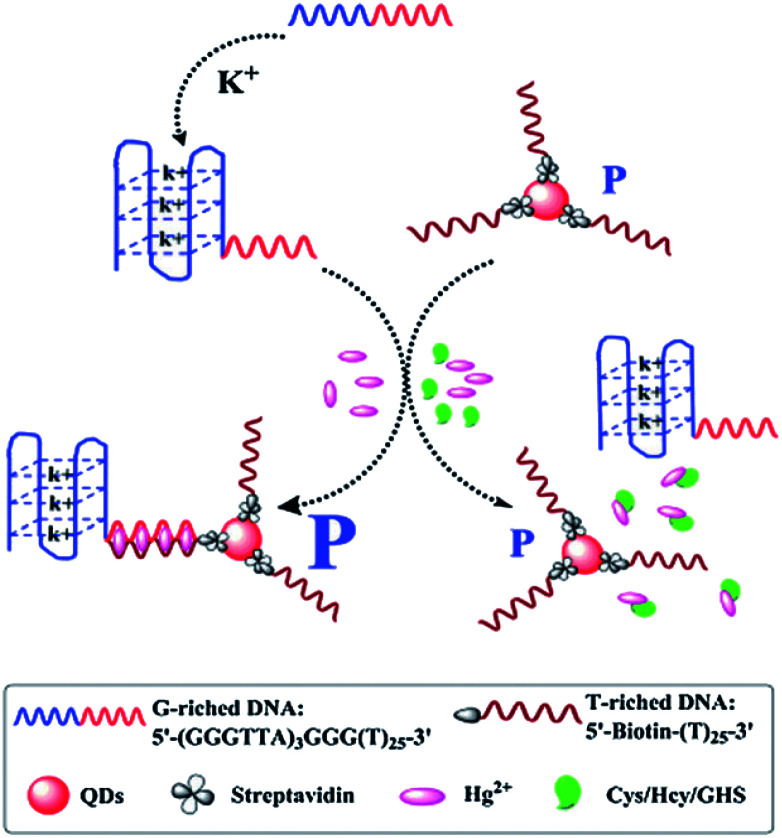
Schematic illustration of the QD FP enhancement homogenous system based on K^+^-mediated G-quadruplex formation for the determination of Hg^2+^ and biothiols. Reprinted with permission from ref. 103. Copyright (2014) Royal Society of Chemistry.

The oligonucleotides enhanced FA/FP assay has been generally researched, which is mainly by changing the structure and sequence of the oligonucleotides chain. Zhao group developed a K^+^-mediated G-quadruplex formation enhancement FP sensor based on CdS–CdTe core–shell quantum dots (QDs) that was constructed for detection of Hg^2+^ and biothiols.^103^ In this study (Fig. 11), CdTe/CdS QD functionalized with T-rich DNA with the affinity of biotin–streptavidin as fluorescence polarization probe, and the 50-guanine-rich sequence of G-rich DNA folded into a G-quadruplex in the presence of potassium ions. In the presence of Hg^2+^, the 3′-thymine-rich sequence of the K^+^-mediated G-quadruplex (GQ-DNA) and QD-T can form large volume complexes through the strong and specific affinity of T–Hg^2+^–T. As a consequence, the K^+^-mediated G-quadruplex leads to dramatic changes in the molecular volume of QDs, thus the FP value of the QDs will significantly increase. Upon the addition of biothiol amino acids, the strong reaction of thiol–Hg^2+^ prohibits the formation of the T–Hg^2+^–T complexes and makes the QD-T and the K^+^-mediated G-quadruplex (GQ-DNA) stay in a free single state. Subsequently, the FP values of the solution greatly decrease. Based on the decrease of FP values, the detection of biothiols can also be realized in this method.

In addition, by taking advantage of the binding-induced change of the TMR−G interaction, Wang *et al.* demonstrated a series of enhanced FA assays for nucleic acid aptamer affinity analysis. These FA strategies allow for analysis of proteins, small molecules, metal ion and DNAzyme activity, showing high sensitivity and selectivity.^5,20,36,44,104–106^ Peyrin *et al.* designed a FA aptamer sensing platform dedicated to small molecule detection that relied on enhanced fluctuations of segmental motion dynamics of the aptamer tracer mediated by an unlabelled, partially complementary oligonucleotide.^107^ Furthermore, by using the accumulation of a lot of DNA in fluorescence indicator-graphene QDs to enhance FA, Hosseini group achieved DNA methyltransferase activity detection.^108^

### 4.6 Other amplification

**Fig. 12:**
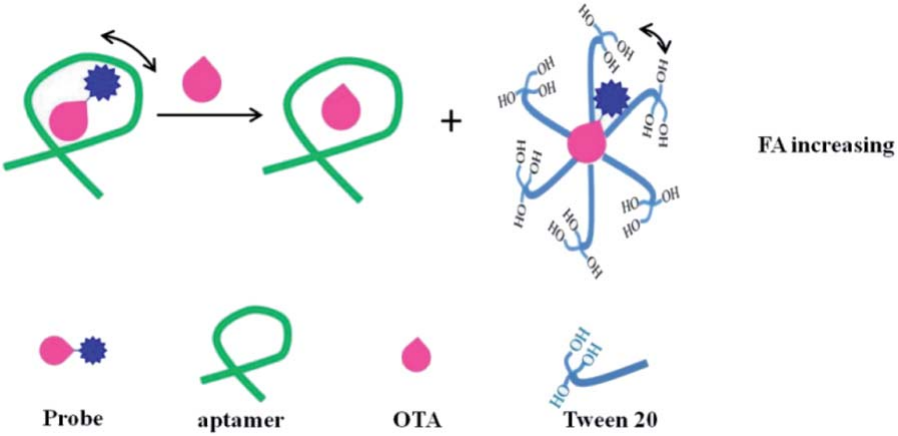
Schematic of competitive aptamer FA assay for the determination of OTA with signal-on responses in a binding buffer that contains a 0.1% Tween 20. Reprinted with permission from ref. 110. Copyright (2020) American Chemical Society.

In addition to the signal amplification methods listed above, the enhancing FA/FP assays by resonance energy transfer (RET) and Tween 20 have been used for biomolecules analysis. Taking advantage of the impact of RET on FP, Ren *et al.* developed a RET-enhanced FA strategy for biomolecules detection.^109^ This method used streptavidin Alexa Fluors 488 conjugated (SA-488), nanogold and biotinylated substrate peptide (biotin–subpeptide) which connects the SA-488 and nanogold to construct a fluorescence variable probe. When the fluorescence molecule conjugates with the nanogold, its volume and mass will increase. Besides, its fluorescence intensity (FI) will be suppressed by the RET, leading to a decrease in its effective concentration. According to the FP principle, both the decrease in concentration and the increase in volume will lead to an increase in FP. When adding target molecules, the conjugation between nanogold and SA-488 will be blocked, resulting in a decreased FP value. Based on the change in FP, trypsin and biotin were detected in this study. In addition, by using Lissamine Rhodamine B labeled ochratoxin A (OTA) as a fluorescence probe, Zhao *et al.* developed a competitive aptamer FA assays for OTA.^110^ In this contribution (Fig. 12), the fluorescent probe can interact with Tween 20. After adding a target, the fluorescent probe shows a higher FA value than that of aptamer–fluorescent probe complex, achieving target sensitive detection through determining the reduced FA value.

## 5. Conclusion and outlook

The measurement ratiometric nature, the homogeneous and high-throughput format, the single dye labeling, and the potential to generate response depending on fluorophore diffusional rotation and/or photochemical changes constitute advantages of the FA/FP technique. Regarding sensitivity, many strategies have been proposed (see Table 1), such as the combination of enzyme-catalyzed target, the application of long-wavelength fluorophores, and signal enhancement approaches using nanomaterials including Ag/Au/SiO_2_ nanoparticles, GO and others. The development of signal amplification methods of FA/FP effectively expanded the application in clinical chemistry and bioassays.

**Table 1:** List of targets currently detected by FA/FP

Targets	Amplification technique	Signal mechanisms	Dynamic ranges	Detection limit	Samples	Ref.
Adenosine	GO amplification	Mass change-based	60–400 μM	22 μM	—	9
Adenosine	Single-stranded DNA binding protein enhancer	Mass change-based	2–50 μM	1 μM	Human serum	11
Adenosine	Phosphodiesterase I and protein amplification	Mass change-based	0.5–1000 μM	500 nM	Cell media	56
Adenosine	GO, nicking enzyme dual amplification	Mass change-based	4 pM to 10 μM	2.0 pM	—	82
Adenosine	Oligonucleotide amplification	Segmental motion change-based	0–6 μM	∼1 μM	—	107
ATP	HCR, protein dual amplification	Mass change-based	0.2–20 μM	100 nM	Cell media, human urine, human serum	12
ATP	Protein amplification	Mass change-based	1–25 μM	0.5 μM	Cell media	28
ATP	GO amplification	Mass change-based	0.5–250 μM	100 nM	Human serum	31
ATP	Digoxin antibody amplification	Mass change-based	10–350 μM	3.7 μM	Serum	111
ATP	Protein, proximity effect amplification	Mass change-based, segmental motion change-based	0.5–500 μM	0.5 μM	Human urine	53
ATP	SiO_2_NPs enhancement	Mass change-based	40 pM to 100 μM	20 pM	—	84
ATP	MoS_2_ nanosheet	Mass change-based	0.3–40 μM	34.4 nM	Human urine	94
ATP	DNA nanosheet amplification	Mass change-based	150–450 μM	43 μM	—	95
Thrombin	GO amplification	Mass change-based	0.5–4 mg L^−1^	0.19 mg L^−1^	—	9
Thrombin	GO, nicking enzyme dual amplification	Mass change-based	2 fM to 200 nM	1 fM	Human plasma	82
Thrombin	TiS_2_ nanosheet, Exo I amplification	Mass change-based	0.05 pM to 100 nM	0.01 pM	Human serum	93
Thrombin	CSDA and PS NP enhancement	Mass change-based	50 aM to 100 pM	28 aM	Human plasma	96
ssDNA	GO amplification	Mass change-based	8–40 nM	4.6 nM	—	9
ssDNA	AuNPs amplification	Mass change-based	—	0.95 nM	—	30
ssDNA	MOF amplification	Mass change-based	0.3–12 nM	0.2 nM	—	32
ssDNA	Exo III amplification	Mass change-based	0.1 fM to 1 nM	83 aM	—	34
ssDNA	AuNPs amplification	Mass change-based	150 pM to 6 nM	73 pM	Serum	60
ssDNA	AuNPs, NSET dual amplification	Fluorescence lifetime-based, mass change-based	—	372 pM	DMEM	66
ssDNA	GO, T7 Exo-assisted amplification	Mass change-based	50–2000 pM	38.6 pM	Human serum	81
ssDNA	SiO_2_NPs amplification	Mass change-based	1–800 nM	0.65 nM	Human serum	85
ssDNA	HCR and SiO_2_NPs amplification	Mass change-based	0–2.5 nM	34 pM	Human serum	86
ssDNA	MOF amplification	Mass change-based	1–20 nM	1 nM	—	89
ssDNA	DNA nanosheet amplification	Mass change-based	10–50 nM	8 nM	—	95
miRNA	Protein amplification	Mass change-based	10 pM to 10 nM	8.5 pM	Cell lysis buffer, cell lysate	29
miRNA	GO and CHA amplification	Mass change-based	0–16 nM	47 pM	Cell extractions	83
miRNA	T7 Exo, polystyrene nanospheres amplification	Mass change-based	0.001–10 nM	0.001 nM	Human serum, cell extractions	97
Hg^2+^	AuNPs amplification	Mass change-based	1 nM to 1 mM	1 nM	River water	65
Hg^2+^	AgNPs amplification	Mass change-based	10–400 nM	6.6 nM	Tap water	67
Hg^2+^	MOF amplification	Mass change-based	20–200 nM	8.66 nM	Tap water	88
Hg^2+^	K^+^-mediated G-quadruplex enhancement	Mass change-based	10–800 nM	8.6 nM	Lake water	103
Cu^2+^	GO and DNAzyme amplification	Mass change-based	1–32 nM	1 nM	—	58
Cu^2+^	DNAzyme self-assembled AuNPs amplification	Mass change-based	0.001–10 μM	∼1 nM	River water	61
Cu^2+^	DNAzyme self-assembled gold nanorods amplification	Mass change-based	8–320 pM	8.40 pM	—	62
Cu^2+^	SiO_2_ NPs amplification	Mass change-based	0.050–2.0 μM	0.0178 μM	River water	87
Pb^2+^	Multiple G bases amplification	Fluorescence lifetime-based	200 pM to 100 nM	100 pM	—	5
Pb^2+^	Phosphate-perylene modification G-quadruplex probes amplification	Segmental motion change-based	25–5000 nM	24.5 nM	Tap water	13
Pb^2+^	Cleavable DNA–protein hybrid molecular beacon amplification	Mass change-based, segmental motion change-based	1–20 nM	0.5 nM	Water samples	57
Pb^2+^	DNAzyme self-assembled AuNPs amplification	Mass change-based	0.001–10 μM	1 nM	River water	61
Ag^+^	AuNPs amplification	Mass change-based	50–750 nM	9.5 nM	Tap water	64
Ag^+^	MnO_2_ nanosheet amplification	Mass change-based	30–240 nM	9.1 nM	Tap water, lake water	92
Cocaine	Protein amplification	Mass change-based	1–100 μM	0.8 μM	Human urine	28
Cocaine	Protein, isothermal exponential amplification	Mass change-based	30 pM to 30 μM	18 pM	Human serum	99
Aflatoxin B_1_	Protein, proximity effect amplification	Mass change-based, segmental motion change-based	60 pM to 125 nM	60 pM	White wine	53
Aflatoxin B_1_	GO amplification	Mass change-based	0.05–5 nM	0.05 nM	Rice extract	78
Aflatoxin B_1_	Protein, isothermal exponential amplification	Mass change-based	0.4 pM to 400 nM	0.24 pM	—	99
Ochratoxin A	Protein amplification	Mass change-based	—	3.6 nM	White wine	51
Ochratoxin A	Protein, proximity effect amplification	Mass change-based, segmental motion change-based	1 nM to 5 μM	1 nM	White wine	53
Cysteine	AgNPs amplification	Mass change-based	20–700 nM	11 nM	—	67
Cysteine	K^+^-mediated G-quadruplex enhancement	Mass change-based	50–2000 nM	9.9 nM	Human urine	103
Ricin B-chain	GO, Exo III-assisted amplification	Mass change-based	1–13.3 μg mL^−1^	400 ng mL^−1^	Orange juice	27
K^+^	GO amplification	Mass change-based	10 μM to 2 mM	1 μM	—	33
DNA–protein interactions	Protein amplification	Mass change-based	—	6.3 nM	—	54
Chloramphenicol	PCR and protein amplification	Mass change-based	0.001–200 nM	0.5 pM	Honey	55
EcoRI endonuclease	AuNPs amplification	Mass change-based	5.0 × 10^−4^ to 10 U mL^−1^	5.0 × 10^−4^ U mL^−1^	—	59
Lac	MEF, Ag_10_NPs amplification	Mass change-based, fluorescence lifetime-based	0.2 ng mL^−1^ to 25 μg mL^−1^	1.25 pM	Milk powder	68
DNA MTase activity	Carbon nanotube signal amplification	Mass change-based	—	1.0 × 10^−4^ U mL^−1^	Human serum	74
Apyrase	Carbon nanoparticle amplification	Mass change-based	0.1–0.5 U μL^−1^	0.05 U μL^−1^	—	75
Lipopolysaccharides (*Salmonella enterica* serotype Typhimurium)	GO amplification	Mass change-based	—	38.7 ng mL^−1^	Sodium chloride injection	79
I^−^	MOF amplification	Mass change-based	0.02–3.0 μM	17.4 nM	Tap water	88
PSA	MOG amplification	Mass change-based	0.5–8 ng mL^−1^	0.33 ng mL^−1^	Human serum	90
Folate receptor	TiS_2_ nanosheet, exo I amplification	Mass change-based	0.01–20 ng mL^−1^	0.003 ng mL^−1^	Human serum	93
DNA glycosylase activity	WS_2_ nanosheet and exo III amplification	Mass change-based	0.00080–0.40 U mL^−1^	∼0.00030 U mL^−1^	Cell extracts	91

At present, most of the developed strategies used aptamer as a target recognition unit. However, only a handful of good nucleic acid aptamers are available. The selection of aptamers binding to small molecules is challenging, partly due to the smaller molecular space for interaction. As a consequence, most of the assays on this topic have repeatedly targeted ATP, adenosine, cocaine, ochratoxin A, and thrombin, because aptamers of high affinity and selectivity are available. To solve this problem, the properties of new technologies should be clear, that is, the approaches for the selection and design of aptamers are needed to generate high-quality aptamers for diverse applications. In addition, almost all the established FA/FP sensors for living cell analysis and reaching the commercial level are still fairly rare because of the complexity of the real biological matrix. Furthermore, the implementation of many amplification mechanisms can further improve both the assay response and analyte detection limit, but at the same time at the expense of the biosensor simplicity in most cases, it will result in increased testing costs. Up to now, most of the developed FA/FP strategies amplify the signal by altering the size of the fluorescent complexes (see Table 1). It is highly demanded to combine with other paths as FA/FP enhancers. Finally, there are few articles focusing on the effect of fluorescence lifetime on FA/FP signal, the research in this direction may further expand the application of FA/FP.

## Conflicts of interest

There are no conflicts to declare.

## Supplementary Material
